# Incidence of surgical site infections after cervical spine surgery: results of a single-center cohort study adhering to multimodal preventive wound control protocol

**DOI:** 10.1007/s00590-022-03379-9

**Published:** 2022-09-14

**Authors:** Vera Spatenkova, Ondrej Bradac, Zuzana Mareckova, Petr Suchomel, Jan Hradil, Eduard Kuriscak, Milada Halacova

**Affiliations:** 1 Neurocenter, Neurointensive Care Unit, Regional Hospital, Husova 357/10, 46063 Liberec, Czech Republic; 2 Department of Anaesthesia and Intensive Care, 3 Medical Faculty, Charles University, Srobarova 50, 100 34 Prague, Czech Republic; 3 Institute of Physiology, First Medical Faculty, Charles University in Prague, Albertov 5, 12800 Prague 2, Czech Republic; 4 Faculty of Health Studies, Technical University of Liberec, Studentská 1402/2, 461 17 Liberec 1, Czech Republic; 5 Department of Neurosurgery, 2nd Faculty of Medicine, Charles University in Prague and Motol University Hospital, V Úvalu 84/1, 150 06 Prague, Czech Republic; 6 Neurocenter, Department of Neurosurgery, Regional Hospital, Husova 357/10, 46063 Liberec, Czech Republic; 7 Department of Clinical Pharmacology, Na Homolce Hospital, Roentgenova 37/2, 150 30 Prague 5, Czech Republic

**Keywords:** Surgical site infection, Incidence of SSI, Preventive infection protocol, Wound complications, Antibiotic prophylaxis

## Abstract

**Purpose:**

The incidence of surgical site infections is considered a relevant indicator of perioperative and postoperative care quality. The aim of this study is to analyze and evaluate SSIs after elective cervical spine surgery under the guidance of our preventive multimodal wound protocol.

**Methods:**

A monocentric observational cohort study analyzed 797 patients who underwent cervical spine surgery from 2005 to 2010 (mean age 51.58 ± 11.74 year, male 56.09%, mean BMI 26.87 ± 4.41, ASA score 1–2 in 81.68% of patients), fulfilling the entry criteria: (1) cervical spine surgery performed by neurosurgeons (degenerative disease 85.19%, trauma 11.04%, tumor 3.76%), (2) elective surgery, (3) postoperative care in our neurointensive care unit. Our preventive wound control protocol management focused mainly on antibiotic prophylaxis, wound hygiene regime, and drainage equipment. All wound complications and surgical site infections were monitored up for 1 year after surgery.

**Results:**

We had only 2 (0.25%) patients with SSI after cervical spine surgery—one organ/space infection (osteomyelitis, primary due to liquorrhea) after anterior surgical approach, and one deep surgical site infection (due to dehiscence) after posterior approach. We had 17 (2.13%) patients with some wound complications (secretion 7, dehiscence 4, hematoma 1, edema 3, and liquorrhea 2) that were not classified as SSI according to the CDC guidelines.

**Conclusion:**

Concerning our study population of patients undergoing elective cervical surgery, with ASA scores 1–2 in 81.68% of our patients, the incidence of SSI was 0.14% after anterior surgical approach, 1.4% after posterior surgical approach, and 0.25% altogether in the referred cohort.

## Introduction

A surgical site infection (SSI) is an infection that occurs after surgery in parts of the body where the surgery takes place. SSIs are defined and classified by the CDC guidelines [1]. SSIs are caused by various factors ranging from those related to patient characteristics, to factors that depend on the hospital and the care provided there [2]. Conditions of patients before surgery such as fever, higher CRP, alcoholism, age, comorbidities, obesity, diabetes mellitus, nutritional status, microbial colonization, coexisting infections, or antibiotics used before surgery belong to the most important factors dependent on the patient [3–5]. The preparation of the patient for surgery, the duration of surgery, the type of surgery or surgical style (surgeon’s competence and technique), the amount of blood lost and transfused, the covering of wounds, hand hygiene and disinfection belong to factors dependent on the hospital, comprising preoperative, intraoperative, and postoperative procedures, exhibit a high degree of preventability regarding the development of SSI [6, 7].

SSIs make up roughly 20% of all hospital-acquired infections [8, 9], and about 5% of patients undergoing surgery develop the SSI that requires an average additional 7 days of hospitalization [10]. Regarding the cervical spine surgery, the pooled incidence of SSI reaches in average 3.4% as showed in one recent meta-analysis study [11], but could reach more favorable values when anterior approach is used (0.1% to 1.6% found in [12–14]).

The preventive multimodal wound control protocol comprises multiple preventive measures that reduce the incidence of SSI [15, 16]. It is an important set of procedures or techniques focusing on the preoperative, intraoperative, and postoperative period, comprising proper surgical hand preparation (before and after all care procedures), patient skin preparation, antibiotic prophylaxis, operating theatre organization, and discipline [6, 7, 17, 18].

An important part of the preventive multimodal wound control protocol is optimal antibiotic prophylaxis focusing on effective dosing and its timing before and during operation, with no continuation after surgery. It is important to mention that excessive or inappropriate use of antibiotics also belongs to well-known problems worsening an epidemiological status due to multidrug-resistant bacteria [19, 20]. For all these reasons, quality control management for the prevention of SSIs must be carefully elaborated and maintained.

The aim of this study was to analyze the incidence of SSIs of elective cervical spine surgery under the guidance of our multimodal preventive wound control protocol. This study is a continuation of our previous single-center study that analyzed the incidence of SSIs after lumbar and thoracic surgery [21].

## Materials and methods

### Study setting

This study was carried at the Neurocenter of the Regional Hospital with 900 beds, analyzing patients who underwent spine surgery over a 6-year period from 2005 to 2010. It examined 797 patients who met the entry criteria: (1) cervical spine surgery performed by neurosurgeons, (2) elective operations, and (3) postoperative care in our eight-bed adult neurointensive care unit (NICU). The exclusion criteria involved: (1) acute surgery, (2) antibiotics used after surgery, and (3) the postoperative period commenced in the standard neurosurgery bed ward. The demographic data of this population, spine diagnoses, and duration of stay in the NICU, along with the values of body mass index (BMI), are shown in Table 1.

**Table 1:** Descriptive statistics of demographic and clinical data of patients with cervical spine surgery

Parameter (*N* = 797)	Unit	%	Mean	Standard deviation	Median	Lower quartile (25%)	Upper quartile (75%)
Age	Year		51.58	11.74	51.00	44.00	58.00
Female	pts	43.91% (350)					
Male	pts	56.09% (447)					
Weight	kg		79.20	15.96	78.00	68.00	90.00
Body mass index (BMI)			26.87	4.41	26,40	23.85	29.60
Spine diagnoses							
Degenerative disease	pts	85.19% (679)					
Trauma	pts	11.04% (88)					
Tumor	pts	3.76% (30)					
Stay							
Neurointensive care unit	Day		1.19	0.96	1.00	1.00	1.00
Standard neurosurgery ward	Day		4.39	4.60	3.00	3.00	4.00
Total hospital stay	Day		6.90	12.11	4.00	4.00	5.00
Diabetes mellitus	pts	9.66% (77)					
Ulcer prophylaxis	pts	14.30% (114)					
Omeprazole	pts	12.67% (101)					

*N*—number of patients, BMI—Body mass index

### Study design

The monocentric observational cohort study was carried out after the approval of the Regional Hospital Ethics Committee for Multicentric Clinical Trials. The data processed were obtained from the prospective database of preventive multimodal nosocomial infection control protocol. The database is maintained since 2001 and contains prospective data related to all parameters collected with respect to monitored nosocomial infections in our NICU as well as other parameters related to patients’ health status.

The following clinical parameters were observed: (1) spine diagnosis; (2) parameters associated with operations—surgery approach and technique, number of vertebrae involved in surgery, reoperations, duration of surgery, use of instrumented fixation; (3) presence of drainage, mechanical ventilation, catheters (artery, central venous), diuresis; (4) administration of corticoids (methylprednisolone, hydrocortisone), transfusions, ulcer prophylaxis, and diabetes mellitus; (5) postoperative care—during the NICU stay evaluated by the Therapeutic Intervention Scoring System (TISS).

The actual physical status and clinical health condition of our patients were evaluated using the American Society of Anesthesiologists (ASA) score and the Acute Physiology and Chronic Health Evaluation (APACHE) II score (supplemented by levels of C-reactive protein (CRP) and BMI values, see Table 2).

**Table 2:** Characteristics of cervical spine operations

Parameter (*N* = 797)	Unit	%	Mean	Standard deviation	Median	Lower quartile (25%)	Upper quartile (75%)
Number of vertebrae							
1	pts	56.71% (452)					
2	pts	34.25% (273)					
3	pts	6.02% (48)					
ASA score 1–2		81.68% (651)					
Reoperation	pts	5.14% (41)					
Time of operation	Minutes		103.72	59.17	90.00	70.00	120.00
Operation approach							
Anterior	pts	88.58% (706)					
Posterior	pts	8.53% (68)					
Instrumented fixation	pts	92.22% (735)					
Drainage	pts	96.24% (767)					
Redon	pts	95.61% (762)					
One drainage	pts	85.82% (684)					
Transfusions	pts	1.13% (9)					
Blood loss	ml		160.61	327.40	50.00	50.00	100.00
Hemoglobin	g/l		130.00	16.86	131.00	121.00	142.00
Corticoids	pts	8.66% (69)					
Methylprednisolone	pts	1.38% (11)					
Hydrocortisone	pts	5.65% (45)					
Antibiotic prophylaxis	pts	98.49% (785)					
1-dose operation 1 V	pts	89.71% (715)					
2-dose operation 2 V	pts	5.77% (46)					
Cefazolin	pts	86.83% (692)					
Clindamycin	pts	10.92% (87)					
Postoperative period							
TISS—NICU admission			57.72	0.76	58.00	58.00	58.00
APACHE II—NICU admission			7.22	3.05	7.00	5.00	9.00
CRP day OP			6.32	14.60	2.00	1.00	5.00
CRP 1 day after OP			23.76	23.02	16.00	8.00	31.00
Mechanical ventilation	pts	1.13% (9)					
Artery catheters	pts	5.14% (41)					
	Day		2.59	2.60	1.00	1.00	3.00
Central venous catheter	pts	2.01% (16)					
	Day		5.94	4.82	5.50	2.00	9.00
Urine catheter	pts	49.69% (396)					
	Day		1.32	1.56	1.00	1.00	1.00

ASA—American Society of Anesthesiologists, NICU—neurointensive care unit, TISS—Therapeutic Intervention Scoring System, APACHE—Acute Physiology and Chronic Health Evaluation, CRP—C-reactive protein, OP—operation

### Multimodal preventive wound control protocol

A crucial part of a preventive multimodal wound control protocol is an optimal antibiotic prophylaxis that focuses on effective dosing and its timing before and during operation, with no continuation after the operation. Cefazolin was the first antibiotic choice, with Clindamycin administered in the case of allergy to beta-lactam antibiotics. Cefazolin was administered 30–60 min before surgery (that is, before the incision, 2 g if body mass was less than 100 kg, otherwise 3 g), and readministered if the surgery lasted more than 4 h, or if the blood loss was greater than 1.5 L. The dose of Clindamycin used was 600 mg, 60 min before surgery, and repeated if surgery was longer than 6 h. (For body weight above 100 kg the dose was 900 mg.)

The hygienic regime consisted of the following measures: (1) hand hygiene before and after all care procedures; (2) surgical face masks, surgical caps, sterile surgical gowns, sterile insertion of systems during invasive procedures; (3) disinfection with soap before entering the operating theatre; (4) rules and procedures for drainage and tubes: single-use products, closed systems only, emphasis on the shortest duration of procedures, minimization of disconnections of used port systems, regular as well as irregular replacements according to the vendor instructions and recommendations; (5) full wound coverage and keeping wounds dry and sterile; (6) full isolation of patients with infections (use of separated patient boxes, enhanced barrier precautions, treating and disposing health-care waste as contaminated, etc.); (7) daily cleaning and disinfecting of surfaces including beds, monitors and other equipment around the bed, door handles and floors.

### Surgical site infections

Surgical site infections were defined according to (1) clinical symptoms, (2) bacterial pathogens, (3) imaging methods, (4) biochemical and hematological laboratory tests, according to the CDC guidelines for SSI [1]. Surgical site infections were followed up for 1 year after surgery.

### Statistical analysis

Statistical analysis was performed using STATISTICA 13.2 software (TIBCO Software Inc., Palo Alto, CA, USA). We evaluated mainly the parameters of descriptive statistics, comprising medians, means, standard deviations (SD), frequencies, percentage, and quartiles of evaluated variables.

## Results

Of the total of 797 patients included in the study, 2 patients had SSI (0.25% of all enrolled individuals, all patients began their postoperative period in the NICU). There was 1 patient with deep surgical site infection and 1 patient with organ/space osteomyelitis. Both patients had preceding wound complications: wound dehiscence in the case of deep surgical site infection and liquorrhea in the case of osteomyelitis. The deep surgical site infection occurred 15 days after the posterior approach to the cervical spine and was caused by Enterobacter cloacae. The osteomyelitis was diagnosed 7 days after the anterior cervical spine surgery and was caused by Staphylococcus aureus. Both patients had diabetes mellitus. The incidence of SSI of the anterior surgical approach was thus 0.14% (one of 706 patients) and of the posterior approach 1.4% (one of 68 patients).

There were 17 patients (2.13%) who had noninfectious wound complications and therefore were not classified as SSI according to the CDC guidelines [1]. Table 3 shows in detail the type of wound complications. They comprised mostly temporary secretion (7 patients), dehiscence (4 patients, 2 cases with dehiscence only, 1 case with dehiscence and secretion, 1 case with dehiscence and hematoma), liquorrhea (2 patients), hematoma (1 patient), and edema (3 patients).

**Table 3:** Wound complications

Parameter (*N* = 797)	Unit	Number	%
Wound complications	pts	17	2.13
Secretion	pts	7	0.88
Dehiscence	pts	2	0.25
Dehiscence with secretion	pts	1	0.13
Dehiscence with hematoma	pts	1	0.13
Hematoma	pts	1	0.13
Edema	pts	3	0.38
Liquorrhea	pts	2	0.25
Surgical site infections	pts	2	0.25
Deep surgical site infections	pts	1	0.125
Organ/Space—Osteomyelitis	pts	1	0.125

## Discussion

The incidence of surgical site infections (SSI) is an indicator of operation care quality and represents an unavoidable risk in any surgery [22]. It reaches 2–11% in all surgical interventions according to the data referred in [23]. It depends both on factors related to the patient characteristics and on factors related to procedures performed on the patient before, during, and after surgery. The incidence of SSI related to spine surgery [24] ranges from 0.7 to 11.9% according to the following data [25–27]. It is often considered an indicator of the sanitary, hygienic, and microbiological regime of a given surgical department and ICU [28], but is also dependent, as mentioned, on the extend and the type of surgery. For example, the Surgical Invasiveness Index (SII)—a composite score comprising the number of vertebrae levels involved, the type of surgery performed at each level, the amount of blood loss, and the duration of surgery—belongs to proven SSI predictors. (An increase in the SII score is associated with a higher incidence of SSI, [29].)

Complications inflicted by SSI should be avoided, although this is not an easy task to achieve. A very important prerequisite in reducing SSIs is a well-managed multimodal preventive protocol focusing on factors that are preventable, including sanitary, hygienic, and microbiological measures, minimizing the contamination of surfaces and wounds with endogenous or hospital pathogens [6]. Established procedural methods in the preoperative and perioperative period confirm a high degree of preventability of SSI [3].

A good clinical outcome of the patient is certainly a primary target; however, there are also implications and concerns for a whole health system. Among them, there are prolonged hospitalizations, increased demands for additional care, increased financial costs, adverse effects on epidemiological status, and antibiotic policy due to the necessity of extensive use of antibiotics. The most demanding aspect of any protocol is not to introduce it, but to adhere to it and keep it working effectively. That must be carried out implicitly by the entire team, focusing equally responsibly and thoroughly on the preoperative, operative, and postoperative period.

In our study, we focused on both anterior and posterior cervical spine operations. We included a relatively large group of patients, summing up 797 individuals who over a six-year period met the study entry criteria (elective cervical spine surgery and the beginning of the postoperative period in NICU). Our results are based on collected data respecting the definition of SSI according to Horan et al. [22] and the CDC SSI guidelines [1]. Over the period of one year following the operation, we observed only 2 cases of SSI, just 0.25% altogether. The reason for such a low incidence of SSI can be seen in the dominance of cases treated for degenerative diseases (85.19%) and possibly in the high prevalence of the anterior approach (88.58%), which has a significantly lower incidence of SSI [30], which corresponds with the SSI incidence in our cohort of patients (0.14% with anterior vs. 1.4% with posterior, or 0.25% comprising both approaches). In one study, analyzing 452 cases, even no SSI was found associated with the anterior spine approach, regardless of the vertebrae level operated [31]. The anterior approach also implies a smaller blood loss (mean 160.61 ± 327.40). This corresponds to the fact that the transfusion was only given to 9 patients, which could have resulted in fewer SSI. (Blood loss and subsequent transfusion are considered to increase the risk of SSI [32], although some works consider blood transfusion to be only a confounding variable that correlates with the duration of surgery [4].) Furthermore, most of our patients had low ASA scores (1–2 in 81.68% of patients), which could also have caused sampling bias toward a healthier population exhibiting fewer SSIs. Among other factors that could be responsible for the low SSI incidence of our patients is the low average age (51.58 years), the relatively low BMI (26.87), and the short total stay in the hospital (6.9 days, 1.19 in the NICU + 4.39 in the standard neurosurgery ward). Furthermore, the fact that all our patients underwent elective surgery may play a role in our low incidence of SSI, since the patient's preparation for surgery, as well as the surgical procedure, could have been performed more cautiously and deliberately compared to acute surgery. Thus, all the facts mentioned could contribute to the observed low incidence of SSI. This can be compared to other studies focusing on cervical surgery referring the incidence of SSI 1.6% ([12]—both approaches combined analyzing 39,893 patients), 1.2% ([13]—a systematic review calculating a pooled incidence analyzing 965,867 patients with anterior approach), or 0.1% (found in [14] analyzing 1015 patients after anterior approach). A certain role in the low incidence of cervical spine SSIs could be attributed to the fact that wounds from spine surgery are located further from the sites or predilected sources of enteral bacterial flora (anus and perineal regions, contaminating surgical wounds due to the patients excretion and related sanitary procedures), an assumption that can partially be supported by a significantly higher SSI incidence of lumbar and thoracic surgery performed at our neurosurgery center (adhering to the same wound prevention protocol, however, with an incidence of SSI reaching 8% [21]).

Risk factors that increase the incidence of SSI include noninfectious wound complications, such as secretion, dehiscence, or liquorrhea. Our wound complications can be seen in Table 3. It is vital to pay special attention to every uninfected wound complication, as they are prone to become infected in a very short time interval. The antibiotic policy, including dosing and scheduling, also plays a very important role in the management of SSIs. With the protocol that is followed properly, it seems that it is not necessary to administer antibiotics in the postoperative period. In our study, only 16 (2.01%) patients received prolonged antibiotic prophylaxis, mainly due to common accidents during surgery (e.g., ruptured gloves). There is a rule in our neurocenter imposing monitoring of all patients after the cervical spine surgery in the NICU to minimize complications until patients are fully stabilized. So, all patients in our study begin their postoperative period in the NICU (this postoperative period was short and lasted 1.19 ± 0.96 days), followed by a stay in the standard neurosurgery bed ward (4.39 ± 4.6 days). That could have reduced the incidence of SSI with surgical wounds carefully monitored and meticulously treated there. However, NICUs, or in general ICUs, are also known to have a generally higher risk of acquiring multiresistant bacterial pathogens [33] compared to staying in the standard surgical ward. Thus, in general, the resulting SSI incidence could be affected in any direction, depending on the actual epidemiological status of a given ICU (especially worrying are the pathogens known as MRSA—Methicillin-resistant Staphylococcus aureus, and extended spectrum β-lactamase (ESBL) producing enterobacteria). At our NICU, the risk of multidrug-resistant bacteria and nosocomial infection was shown to be low [34]. A routine surgical technique performed by our team that focuses on a narrow spectrum of surgery types could also be taken into account to explain the lower incidence of SSI. In our NICU, we pay special attention to comply with the rules of our protocol, keeping the wounds dry and completely covered, and trying not to prolong the antibiotic prophylaxis unnecessarily. Since one of our main goals was to evaluate the validity of our multimodal preventive wound control protocol, having only 2 SSIs from 797 patients (0.25%) indicates that our protocol is effective and contributes significantly to the minimalization of SSIs.

Our study has several limitations. Low and favorable ASA (81.68% of patients had ASA scores 1–2) can be considered as one of them, resulting in the lack of SSI data on the incidence of SSI in patients with higher, possibly more frequent ASA scores. A higher ASA score directly increases the incidence of wound complications and implies a prolonged stay in hospital, thus contributing additionally to the higher incidence of SSI. We also did not assign our patients to subgroups of smokers and nonsmokers, the state of nutrition on SIS incidence was also not studied in detail, as well as we were not able to extract other risk factors of SIS from our data. The main reason behind that is that in the presented study we had only 2 cases of SSI from 797 patients enrolled in the study, which prevented meaningful comparison of these two incommensurable groups of patients (case group comprising 2 patients vs. 795 patients that would make a control group), and hindered any reasonable comparative analysis of SSI risk factors within our group of patients. Since this is an observational, single-center study, we were also not able (for ethical as well as operational reasons) to compare two groups of patients, one under our current multimodal preventive wound protocol, with another one adhering to a different protocol. We are fully aware that the low incidence of SSI in this study is also partially a consequence of other factors that are not fully related to the parameters of our preventive wound protocol, factors already discussed in previous paragraphs, related primarily to the characteristics of the population involved in this study.

## Conclusions

The incidence of surgical site infection can be kept considerably low in the cervical spine surgery once the proper multimodal preventive wound control protocol is introduced and maintained. (In our case, the incidence of SSI after anterior surgical approach was 0.14%, after posterior approach 1.4%, and for both approaches 0.25%.) These values pertain to specific group of patients observed in our hospital who underwent the elective cervical surgery, had an average age close to 51 years, a low ASA score (1–2 in 81.68% of patients), and an indication for surgery due to degenerative disorders (85.19%).

## Data Availability

The datasets used and/or analyzed during the current study are available from the corresponding author on reasonable request. The data are not publicly available because they contain information that could compromise participant privacy. Incidence of surgical site infections after cervical spine surgery: Results of a single-center cohort study adhering to multimodal preventive wound control protocol.
